# Proteomics profiling of inflammatory responses to elexacaftor/tezacaftor/ivacaftor in cystic fibrosis

**DOI:** 10.3389/fimmu.2025.1486784

**Published:** 2025-01-28

**Authors:** Hazel Ozuna, Dinesh Bojja, Santiago Partida-Sanchez, Luanne Hall-Stoodley, Amal Amer, Rodney D. Britt, Shahid Sheikh, David A. Frank, Weiyuan Wang, Bum-Yong Kang, Irina Miralda, Samantha L. Durfey, Benjamin T. Kopp

**Affiliations:** ^1^ Center for Cystic Fibrosis and Airways Disease Research (CF-AIR), Emory University School of Medicine, Atlanta, GA, United States; ^2^ Division of Pulmonology, Asthma, Cystic Fibrosis, and Sleep, Department of Pediatrics, Emory University School of Medicine, Atlanta, GA, United States; ^3^ Yale University, New Haven, CT, United States; ^4^ The Abigail Wexner Research Institute at Nationwide Children’s Hospital, Columbus, OH, United States; ^5^ Department of Microbial Infection and Immunity, The Ohio State University, Columbus, OH, United States; ^6^ Department of Pediatrics, The Ohio State University, Columbus, OH, United States; ^7^ Winship Cancer Institute, Emory University School of Medicine, Atlanta, GA, United States

**Keywords:** cystic fibrosis, inflammation, proteomics, modulators, NF-κB

## Abstract

**Background:**

CFTR modulator therapies have positive clinical outcomes, yet chronic inflammation and bacterial infections persist in people with CF (pwCF). How elexacaftor–tezacaftor–ivacaftor (ETI) fails to improve innate immune signaling responsible for bacterial clearance and inflammation resolution remains unknown.

**Methods:**

We used an unbiased proteomics approach to measure the effect of ETI on inflammatory proteins. Plasma from 20 pediatric pwCF and 20 non-CF (NCF) was collected during routine examination and 3 months after ETI initiation. Protein screening was performed with an inflammation panel (Target 96, Olink^®^). Bioinformatics analysis was used to determine changes in protein expression.

**Results:**

There were significantly fewer pulmonary exacerbations after ETI initiation, along with sustained improvement in lung function and reduced bacterial colonization. Unpaired analysis of CF pre-ETI and NCF resulted in 34 significantly different proteins. Of these, CCL20, MMP-10, EN-RAGE, and AXIN1 had a log_2_ fold change of 1.2 or more. There was a modest shift in overall CF protein profiles post-ETI toward the NCF cluster. Unpaired analysis of protein differential expression between NCF and CF post-ETI identified a total of 24 proteins significantly impacted by ETI therapy (*p*-value ≤ 0.05), with only CCL20 having a log_2_ fold change higher than 1.2. Paired analysis (CF pre- and CF post-ETI) of differential protein expression demonstrated significant expression changes of MMP-10, EN-RAGE, and IL-17A. Pathway analysis identified significantly impacted pathways such as the NF-κB pathway.

**Conclusion:**

This study showed that ETI in a pediatric cohort had a modest effect on several inflammatory proteins with potential as biomarkers. Pathways significantly impacted by ETI can be further studied for future therapies to combat persistent inflammation and dysregulated immunity.

## Introduction

Cystic fibrosis (CF) is a progressive, multi-organ, genetic disease caused by dysfunctional CF transmembrane conductance regulator (CFTR) (1). CFTR-dependent defects cause airflow obstruction and excessive, inflammatory responses characterized by an influx of neutrophils that release elastase, high amounts of nucleic acids, and cytosol matrix-degrading proteins. Combined with defective phagocytosis of pathogens and inflammatory debris, these deficiencies result in progressive tissue damage (2–6). Additionally, the accumulation of thickened mucus secretions creates the perfect environment to harbor bacteria. Alterations in the CF lung microbiome cause decreased bacterial diversity dominated by methicillin-resistant *Staphylococcus aureus* (MRSA) and *Pseudomonas aeruginosa* (7, 8). However, people with CF (pwCF) fail to mount effective and controlled immunity-driven clearance of infections, perpetuating the cycle of infection and exaggerated inflammatory responses that lead to additional pathogen-derived lung damage (9–11).

The arrival of the CFTR modulator therapy elexacaftor/tezacaftor/ivacaftor (ETI) has proven effective for pwCF with eligible CFTR variants. Studies show that ETI helps increase lung function and decrease pulmonary exacerbations (12, 13). Increases in appetite (14) and weight (15–19) were accompanied by improved hyperglycemia, and glycemic variability and increased endocrine pancreatic function were shown post-ETI (20–22). Nevertheless, emerging evidence suggests that CF immune dysfunction persists despite partial CFTR channel restoration with ETI (23–31). Consequently, we sought to use an unbiased proteomics approach to determine whether ETI therapy can change inflammatory protein expression. The findings from this study can help inform on potential limitations to ETI therapy in dampening proinflammatory responses and identify new targets for the development of future CF therapies that will aid against persistent infection and inflammation.

## Methods

### Human subjects

Human subjects were recruited as approved by the Institutional Review Board of Nationwide Children’s Hospital (IRB16-01020) and Emory University (STUDY00004965). All experiments were performed in accordance with relevant guidelines and regulations. Study subjects were provided written informed consent for procedures if of legal age, and children provided written informed assent and a parent or guardian of any child participant provided informed consent on their behalf. From clinic visits, clinical information was recorded into a REDCap database.

### Proteomics processing: Olink^®^ assay

**Figure 1:**
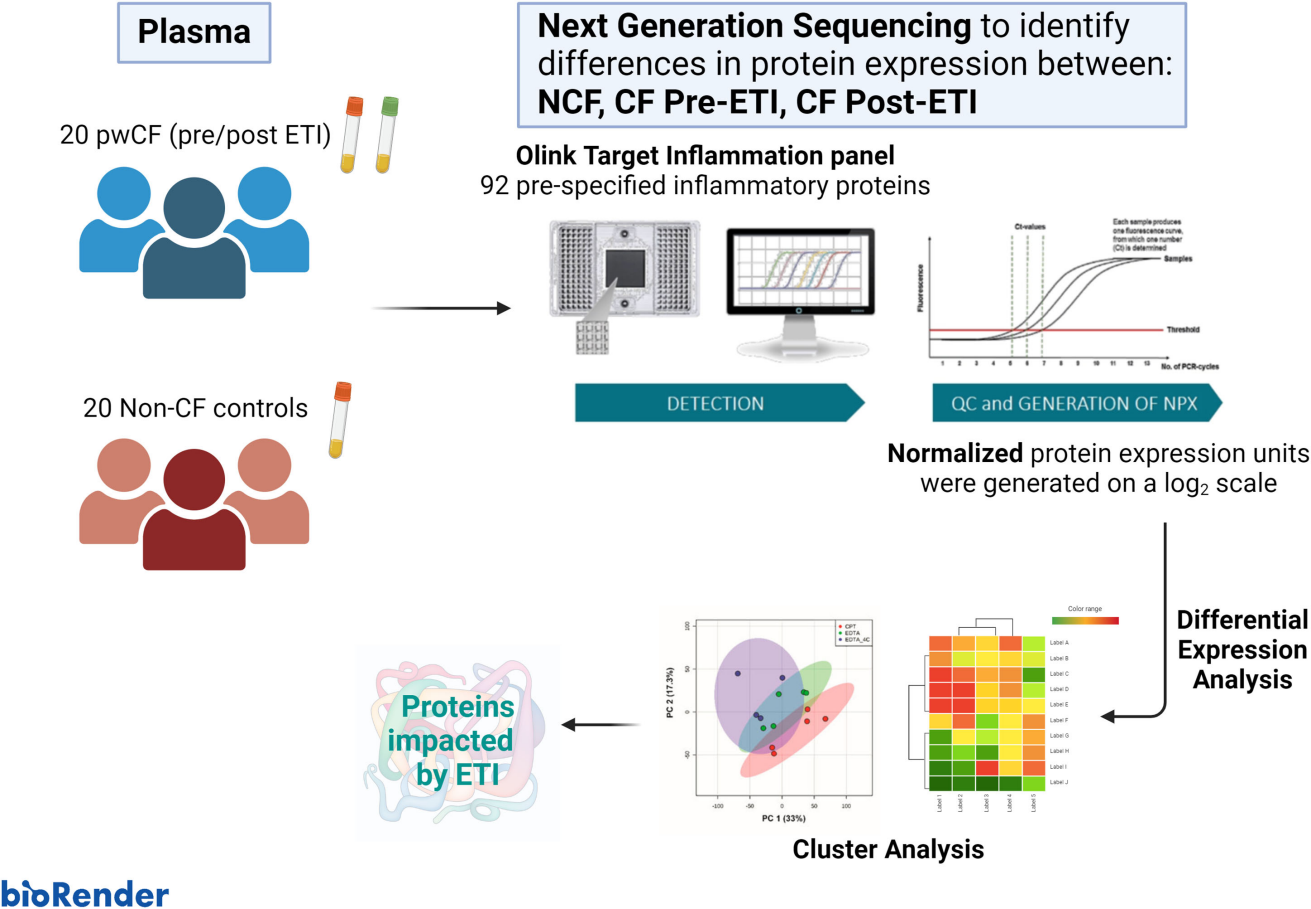
Graphical abstract. The cohort study consisted of 20 non-CF (NCF) controls and 20 people with cystic fibrosis (pwCF). Blood was collected from NCF participants and pwCF before and after elexacaftor/tezacaftor/ivacaftor (ETI) therapy. Plasma was isolated and used for next-generation sequencing to identify differences in protein expression using the OLINK^®^ Target Inflammation panel. Differential expression analysis and downstream cluster analysis were performed using ExpressAnalyst and MetaboAnalyst.

**Figure 1** depicts the process followed in our study. Blood samples were taken during routine CF clinical examinations pre- and 3 months post-ETI therapy. Blood was immediately processed for plasma separation and frozen at −80°C until further processing. Samples were sent for analysis using the Olink^®^ Target 96 Inflammation Proteomics platform (Olink^®^ Bioscience, Boston), which measures 92 prespecified inflammatory proteins in as little as 1 μL of plasma. The Olink^®^ platform is based on the proximity extension assay (PEA) technology, where 92 oligonucleotide-labeled antibody probe pairs are allowed to bind to their respective target proteins, if present in the sample (32, 33). Olink^®^ uses a next-generation sequencing (NGS) approach, consisting of a PCR reporter sequence formed by a proximity-dependent DNA polymerization event. This was amplified and subsequently detected and quantified using real-time PCR. Internal and external controls are included in each sample for quality control of each step in the Olink^®^ protocol (two immunoreaction, extension, and amplification/detection controls). Samples that did not pass quality control were excluded from the analysis (*n* = 1). Data then underwent preprocessing normalization whereby values are set relative to a correction factor (Cq-value) to determine normalized protein expression (NPX) units generated on a log_2_ scale.

### Data and statistical analysis

Differential expression analysis (DEA) pairwise comparisons of NPX (matched to metadata) were performed using a Limma statistical method. The study design for DEA defined “group” as the primary factor, and no secondary factor (and blocking factor) was selected on ExpressAnalyst [https://www.expressanalyst.ca (34)]. Specific parameters consisted of a one-way ANOVA analysis with pairwise comparisons of non-CF (NCF) and pwCF before and after ETI matched to metadata of interest. The metadata collected and used for this analysis were as follows: study groups (groups: CF pre-ETI, CF post-ETI, NCF), responder to therapy (yes/no) based on ≥3% change in forced expiratory volume in 1 s (FEV_1_) and 0.3 body mass index (BMI) or >10% FEV_1_ alone (23), micro clearance of bacteria on clinical respiratory cultures (yes/no), prior CFTR modulator therapy (yes/no), results of the sweat chloride test (negative/indeterminate/positive), and genotype (heterozygous F508del, homozygous F508del, other). Differential expression values were used for different downstream cluster analyses to determine principal component analysis (PCA) variation profiles (https://www.metaboanalyst.ca), to create heatmaps of hierarchical clustering of differential NPX matched to metadata (ExpressAnalyst), and a supervised correlation heatmap of differential NPX (https://appyters.maayanlab.cloud/#/). Differential NPX data comparison (ExpressAnalyst) between clinical states was organized for visualization as volcano plots using SRplots [http://www.bioinformatics.com.cn/srplot (35)]. Differential NPX differences were considered significant with an adjusted *p*-value ≤0.05 and log_2_ fold change of at least 1.2 or higher. Regulation of biological processes was determined using MetaCore enrichment analysis to identify pathways, process networks, and gene ontology (GO) processes impacted by ETI. Top biological processes were determined using GO pathway analysis on ExpressAnalyst and visually organized on SRplot. Clinical outcomes were compared using paired *t*-test statistics via GraphPad Prism v10.1. Significant differences in NPX were determined using a one-way ANOVA with an *α* cutoff of 0.05, and *post-hoc* corrections are indicated in the figure legends.

### Receiver operating characteristic analysis

Variable importance projection scores for predictive modeling of inflammatory proteins that differentiated between clinical states were obtained with a multivariate receiver operating characteristic (ROC) analysis on MetaboAnalyst. We performed automated important feature identification and performance evaluation. ROC curves were generated with a Monte Carlo cross-validation (MCCV) with balanced subsampling. A support vector machine (SVM) multivariate algorithm was used as the classification method and univariate area under the receiver operating characteristic (AUROC) was used for average importance ranking.

### Integrated functional quantitation assay of modulation of transcriptional pathways

To assess the effect of plasma from CF patients on the activation of transcription factors that control key physiologic pathways, STAT1-, STAT3-, STAT5-, and NF-κB-luciferase reporter cell lines were used (36, 37). These cell lines were cultured in DMEM with 10% FBS, 1% penicillin–streptomycin, and 0.2% Normocin. One day before the assay, cells were split and plated in a 96-well plate at 8,000 cells/well, in a volume of 50 µL per well. Following a 24-h incubation at 37°C, 30 µL of plasma from CF patients was added to each well, with four replicates per condition. Following a 30-min preincubation, each well was treated with either 20 µL of culture media as a control or stimulated with 20 µL of 1 ng/mL of IFN-γ for the STAT1-luciferase cells, 1 ng/mL of oncostatin M for the STAT3-luciferase cells, 10 ng/mL of prolactin for the STAT5-luciferase cells, and 10 ng/mL of TNF-α for the NF-κB-luciferase cells. Oncostatin M (300-10), IFN-γ (300-02), prolactin (100-07), and TNF-α (300-01A) were obtained from PeproTech US (Cranbury, NJ). Following a 6-h incubation at 37°C, luciferase activity was quantified using the Bright-Glo Luciferase Assay system (Promega Madison, USA) and a Luminoskan Ascent Luminometer (Labsystems, Vantaa, Finland).

## Results

### Demographics

Twenty pediatric pwCF and 20 healthy controls without CF were enrolled in the study. The basic demographics of the participants are listed in **Table 1** including 1-year post-ETI data. Only 30% of pwCF had been on prior CFTR modulators. Pulmonary exacerbations were significantly decreased in the year after ETI initiation along with a significant sustained improvement at 1 year in lung function (FEV_1_) and reduced bacterial colonization. BMI *z* scores were not significantly changed. Individual metadata at baseline, 3-month, and 1-year follow-up (CF only) of the participants are listed in **Supplementary Table 1**.

**Table 1:** Demographics.

	CF pre-ETI	CF post-ETI	Non-CF	p-value
Age (years)	13.9 ± 5.9	–	24.4 ± 2.1	–
Female	45%	–	50%	–
White	95%	–	95%	–
Pancreatic insufficient	90%	–	–	–
Prior CFTR modulators	30%	–	–	–
PEx per year	1.6 ± 1.6	0.6 ± 0.9	–	0.007
FEV_1_	92.1 ± 11.8	99.4 ± 12.3	–	0.007
BMI z score	−0.04 ± 0.8	0.03 ± 0.8	–	0.25
*S. aureus*	90%	70%	–	0.04
*P. aeruginosa*	15%	0%	–	0.08
Fungal infection	10%	5%	–	0.33

PEx, pulmonary exacerbations; FEV_1_, forced respiratory volume in 1 s; BMI, body mass index.

### The impact of ETI therapy on the expression of inflammatory proteins was moderate

**Figure 2:**
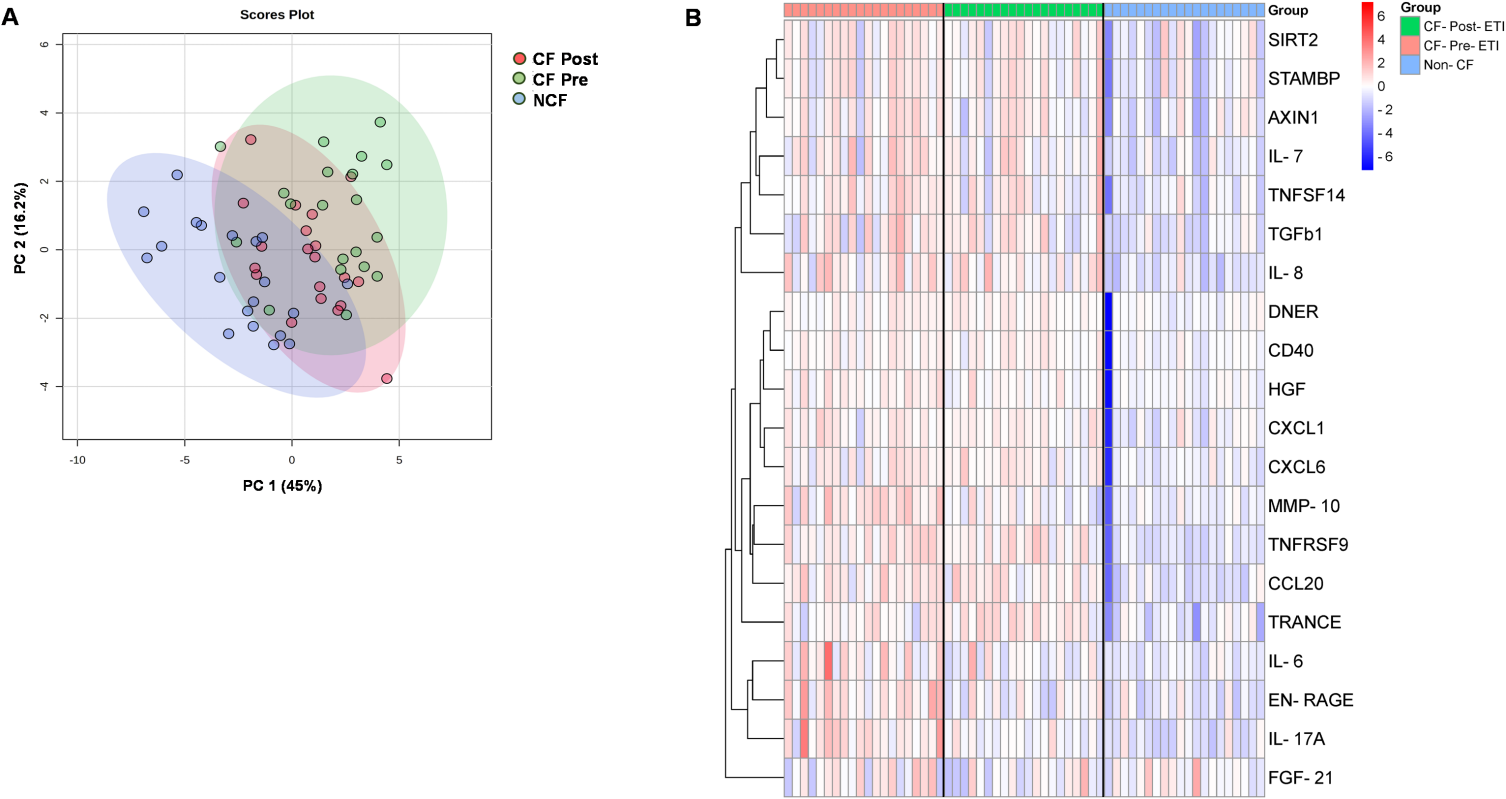
Protein expression profiles of inflammatory proteins. **(A)** Global principal component analysis (PCA) of proteomics profile between non-CF (NCF) controls, CF pre-ETI, and CF post-ETI. **(B)** Partial heatmap of abundance variation profile for each group, by hierarchical clustering with complete linkage, with distance determined by the Euclidean method.

After Olink^®^ quality control analysis (described in the Methods and **Figure 1**), a total of 74 of 92 proteins were detected in >75% of plasma samples. Global clustering protein profiles are shown in **Figure 2**. Principal component analysis (PCA) was used to determine the proteome dynamics among the studied groups: pwCF before (CF pre), after (CF post) ETI therapy, and NCF controls. While the PCA showed distinct clustering of each group, the CF post-ETI group clustered between CF pre-ETI and NCF, with ~50% of CF post-ETI samples shifting toward an NCF profile (**Figure 2A**). A partial heatmap of overall protein expression profiles was generated using ward clustering (**Figure 2B**, see **Supplementary Figure 1** for a complete heatmap). The heatmap showed heterogeneity among protein expression in the population; however, a modest effect on several inflammatory proteins was observed.

**Figure 3:**
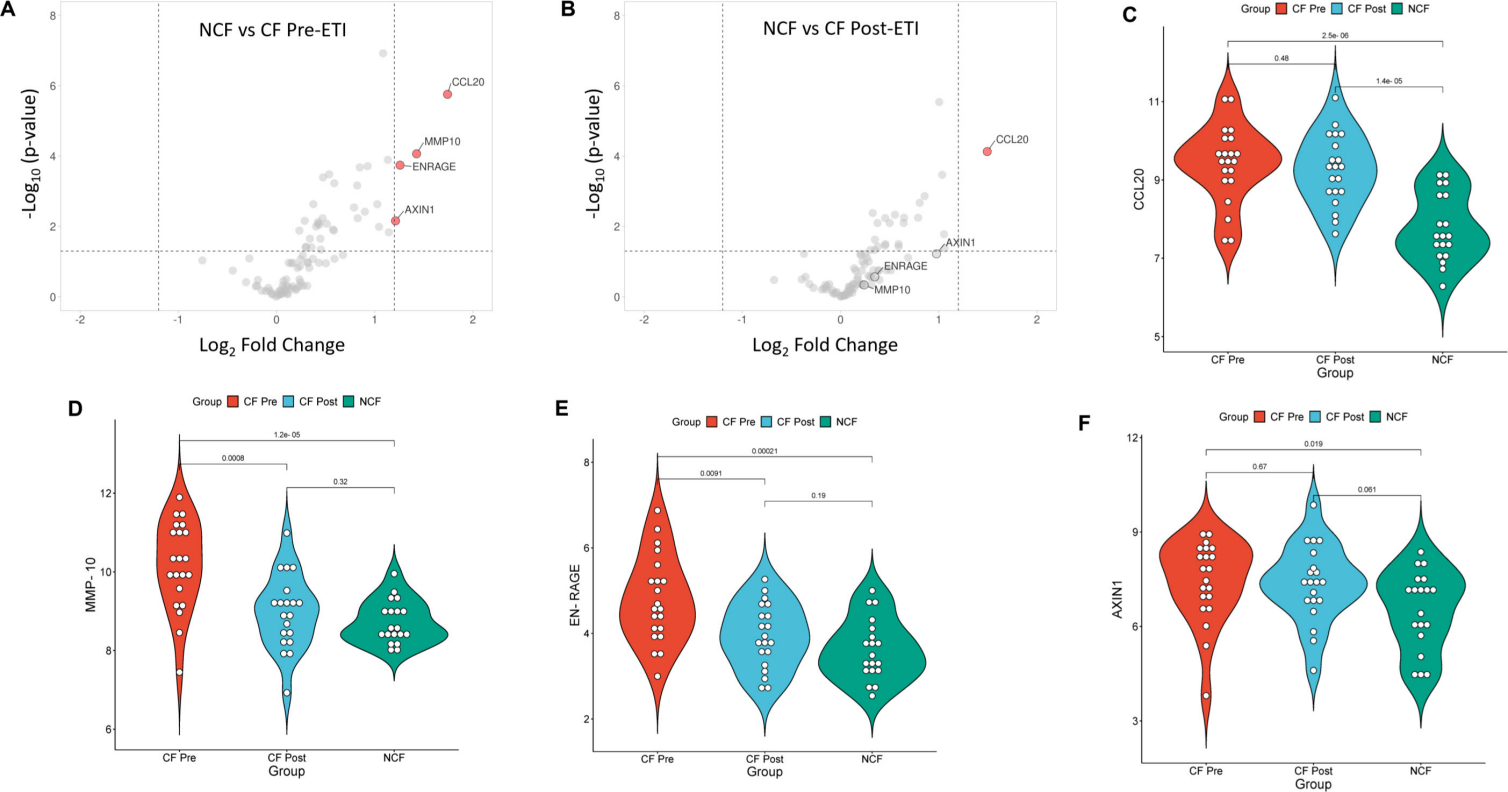
Unpaired analysis of differentially expressed proteins among the study groups. Comparison of the differential expression of NCF vs. CF pre-ETI identified four proteins and NCF vs. CF post-ETI identified one protein significantly impacted by ETI. **(A, B)** Volcano plots present upregulated proteins with an adjusted *p ≤*0.05 and a fold change of 1.2 or more. Statistical significance of protein expression for **(C)** CCL20, **(D)** MMP-10, **(E)** EN-RAGE, and **(F)** AXIN1. One-way ANOVA with Bonferroni correction *p* ≤ 0.05 FC ≥ 1.2 *N* =20 for each group.

**Figure 4:**
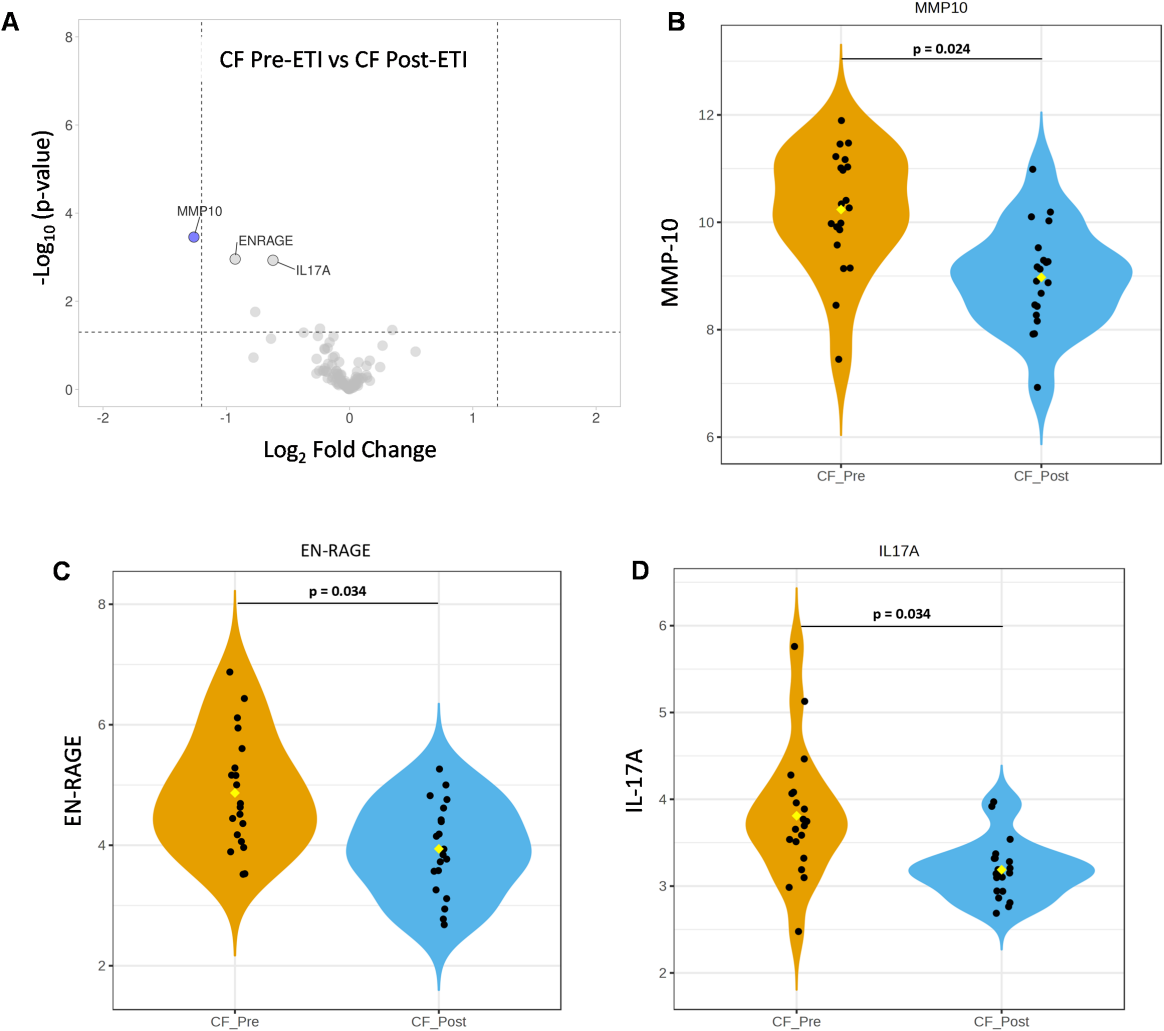
Paired analysis of elexacaftor/tezacaftor/ivacaftor (ETI) impact on the expression of inflammatory proteins. Comparison of differential expression of CF pre-ETI vs. CF post-ETI identified one protein significantly impacted by ETI. **(A)** Volcano plot presents that MMP-10 was significantly downregulated post-ETI (blue = downregulated). Statistical significance of protein expression for **(B)** MMP-10, **(C)** EN-RAGE, and **(D)** IL-17A. One-way ANOVA with Bonferroni correction *p* ≤ 0.05 FC ≥ 1.2 *N* =20 for each group.

To further assess protein expression changes, NPX data were used for protein DEA. We screened for proteins with significant changes in expression using both an adjusted *p*-value of 0.05 or less and a stringent log_2_ fold change of at least 1.2 or higher. Unpaired analysis of NCF vs. CF pre-ETI resulted in 34 proteins with an adjusted *p*-value ≤0.05 and log_2_ fold changes ranging between 0.24 and 1.7 (**Supplementary Table 2**). Of these proteins, we identified four proteins with log_2_ FC ≥1.2, namely, CCL20 (1.7 FC), MMP-10 (1.5 FC), EN-RAGE (1.3 FC), and AXIN1 (1.2 FC), which were upregulated compared to NCF (**Figure 3A**). Unpaired analysis of NCF vs. CF post-ETI resulted in 24 proteins with adjusted *p*-value ≤0.05 and log_2_ FC range of 0.30 to 1.5 (**Supplementary Table 3**). CCL20 was the only protein that remained upregulated after ETI when compared to NCF, at an FC of 1.5 (**Figure 3B**). In **Figure 3C**, it can be appreciated that although CCL20 decreased, the change was small and it remained upregulated. On the other hand, MMP-10 (**Figure 3D**) and EN-RAGE (**Figure 3E**) violin plots are comparable to NCF and distinguishable from CF pre-ETI. Meanwhile, the AXIN1 (**Figure 3F**) violin plot shows that CF post-ETI remains intermediate between CF pre-ETI and NCF. A paired analysis of CF pre-ETI vs. CF post-ETI identified three proteins (**Supplementary Table 4**) whose expression was significantly changed with adjusted *p*-value ≤0.05 and log_2_ fold changes ranging between 0.62 and 1.26. MMP10 with a 1.26 log_2_ FC was downregulated post-ETI (**Figures 4A, B**). However, EN-RAGE (0.93 FC) and IL-17A (0.62 FC) expression decreased and their adjusted *p*-value was found to be statistically significant (**Figures 4C, D**). Combined, we identified modest changes in overall proteomics profiles post-ETI.

### Assessment of diagnostic importance of inflammatory proteins impacted by ETI

**Figure 5:**
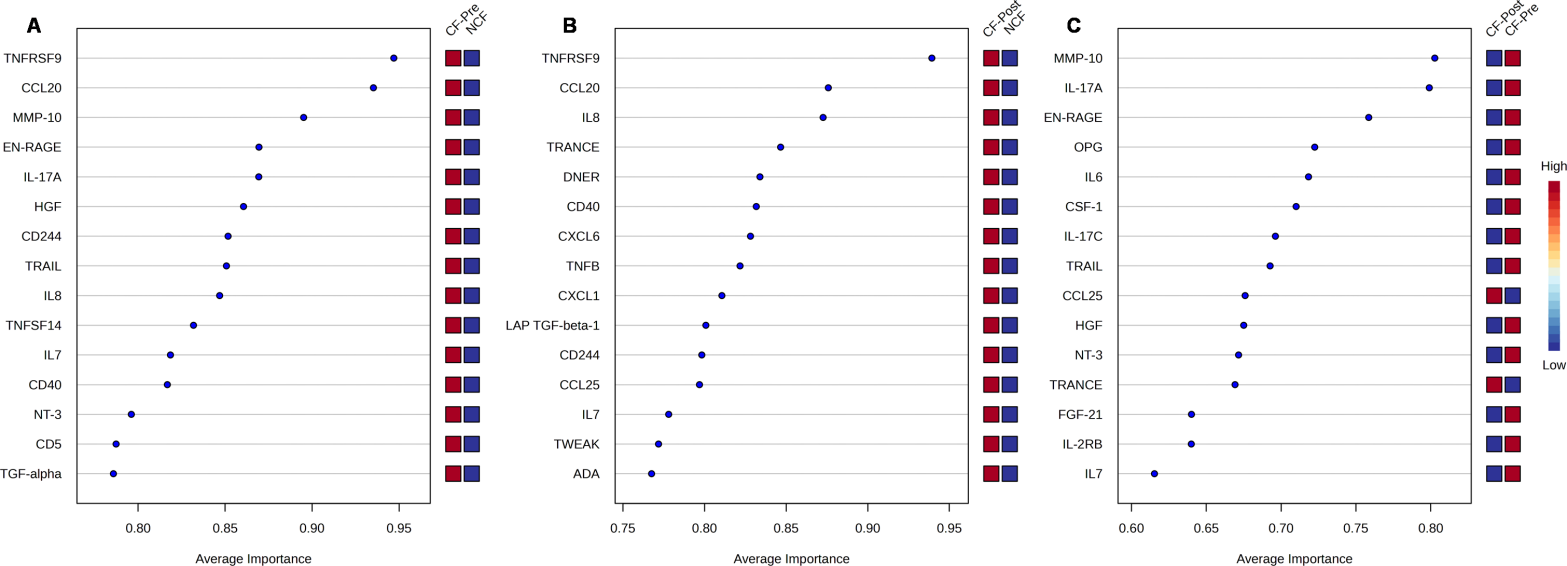
Multivariate receiver operating characteristic (ROC) analysis of potential biomarkers. Average importance (*x*-axis) vs. protein expression (right *y*-axis) for the top 15 proteins of **(A)** cystic fibrosis (CF) prior elexacaftor/tezacaftor/ivacaftor (ETI) therapy vs. non-CF (NCF), **(B)** CF post-ETI therapy vs. NCF, and **(C)** CF prior ETI vs. CF post-ETI. The right *y*-axis shows the levels of expression for a given protein (red = high, blue = low).

To identify which proteins impacted by ETI hold potential and therapeutic analysis, we performed a multivariate receiver operating characteristic (ROC) analysis in all three study groups. ROC ranks feature by average importance, which is a global measure of the ability of a test to discriminate whether a specific condition is present or not and find the best combination of parameters to predict group membership. ROC plots with area under the curve (AUC) values and confidence intervals for each model considered are summarized in **Supplementary Figure 2**. In **Figure 5**, we show the resulting top 15 proteins, ranked by average of importance (AI) between pwCF before and after ETI therapy; the closer the value is to 1, the more important the protein is considered. Most proteins were highly expressed (indicated in red squares) in pwCF before ETI compared to NCF with TNFRSF9 and CCL20 being the highest-ranked (**Figure 5A**) proteins in AI. Supporting our observation in **Figures 3**, **4**, the proteins MMP-10, EN-RAGE, and IL-17A were also highly ranked in AI. Because TNFRSF9 and IL-17A had an AI higher than 0.8, it suggests that their FC can be of biological significance. A similar trend was observed when pwCF post-ETI were compared to NCF, where TNFRSF9 and CCL20 remained the highest-ranked proteins (**Figure 5B**). Additionally, all plotted proteins remained upregulated after ETI when compared to NCF. In contrast, when paired CF samples were compared, proteins CCL25 and TRANCE (TNFSF11), whose low protein expression is considered protective, were found to be upregulated after ETI compared to CF pre-ETI (**Figure 5C**). All the other plotted proteins, including the top AI-ranked proteins MMP-10, IL-17A, and EN-RAGE, were decreased post-ETI (**Figure 5C**). Furthermore, FGF21 is found to be downregulated post-ETI when compared to CF pre-ETI (**Figure 5C**). During fasting, FGF21 is released in the liver and was crucial for the regulation of glucose and lipid metabolism and energy homeostasis. A low expression of this protein is protective, in agreement with our results. On the other hand, we identified CCL11, ADA, FGF19, CCL25, TNFSF11, and EIF4EBP1 (**Supplementary Tables 2**-**4**), whose low protein expression was considered protective, yet our data demonstrated that their expression remained upregulated not just in comparison to NCF but also CF pre-ETI. In conclusion, ROC analysis allowed us to support our initial observations and evaluate other proteins that fall out of the FC cutoff but can have biological significance and present potential topics for future research in CF.

CF pre- and CF post-ETI samples were further stratified by microbial clearance to evaluate whether bacterial eradication drives the reduction of proinflammatory proteins in people with CF post-ETI therapy. Among the 20 CF pre-ETI samples, 3 showed no evidence of infection, increasing to 6 samples post-ETI therapy with no bacterial infection. A closer analysis of these 6 matched samples revealed a total of 36 inflammatory proteins with a fold change of ≥1 post-ETI (**Supplementary Figures 3**, **4**). However, these changes were not statistically significant (*p* ≤ 0.05). This aligns with the modest gene expression changes observed across the full set of 20 samples when comparing CF pre- and CF post-ETI groups. Therefore, we conclude that the decreased proinflammatory profile cannot be attributed solely to the increased bacterial eradication in some pwCF post-ETI therapy.

CF pre- and CF post-ETI groups were also stratified based on ΔppFEV1% (**Supplementary Figure 5**). We observed a strong negative correlation between increased FEV1 and the expression of MMP-10 (*r* = −0.70) and IL-17A (*r* = −0.67). Additionally, the decreased expression of EN-RAGE also correlated with increased FEV1, though at a lower magnitude (*r* = −0.38). These findings further supported the potential of MMP-10 and IL-17A for biomarkers in CF.

### Significant canonical pathways were moderately impacted by ETI

To evaluate the relationships between the proteins identified during unpaired analysis in signaling pathways, we performed a Spearman rank correlation analysis. As mentioned above, unpaired analysis of NCF vs. CF pre-ETI identified 34 differentially expressed proteins and that of NCF vs. CF post-ETI revealed 24 differentially expressed proteins. Correlation analysis among inflammatory proteins revealed three clusters (**Supplementary Figure 6A**) displaying strong positive correlations. The first correlation cluster included members of the TNF superfamily: TRANCE, TRAIL, TNF, and IL-12B, a cytokine expressed by activated macrophages. The second cluster included ST1A1, AXIN1, and SIRT2, proteins previously associated with fibrosis (38–40). Also, in the second cluster were STAMBP, TNFSF14, IL-7, CD40, CD244, and LAP TGFβ_1_, which are linked to impaired lung function and damage. TGFβ is considered a genetic modifier of CF and a severe lung pathology biomarker (41–45). The neutrophil chemokines CXCL1 and CXCL6 are included in the second cluster as well and are released by lung epithelial cells and found in high levels in CF (46, 47). Lastly, the third cluster included IL-17A, MMP-10, HGF, EN-RAGE, IL-6, and TGF-α, all proteins involved in the NF-κB signaling pathway.

Furthermore, we performed gene set enrichment analysis (GSEA) using the GO database to confirm the biological pathways that these proteins are involved in. Pathway analysis resulted in 380 hits for the biological process (BP) domain, 27 hits for molecular functions (MFs), and 35 hits for cellular components (CCs). Of these, 13 BPs, 3 MFs, and 1 CC had a significant enrichment score (**Supplementary Table 5**). Biological processes that were found to be significantly enriched and are of interest to CF are involved in the regulation of intracellular transport, protein import into the nucleus, cell-to-cell signaling, regulation of lipid and cellular metabolic processes, and regulation of defense response, among others (**Supplementary Figure 6B**, top). MFs significantly enriched and of relevance to CF include functions related to transition metal ion binding, zinc ion binding, and cation binding (**Supplementary Figure 6B**, middle). Significantly enriched CCs include receptor complex components (**Supplementary Figure 6B**, bottom).

**Figure 6:**
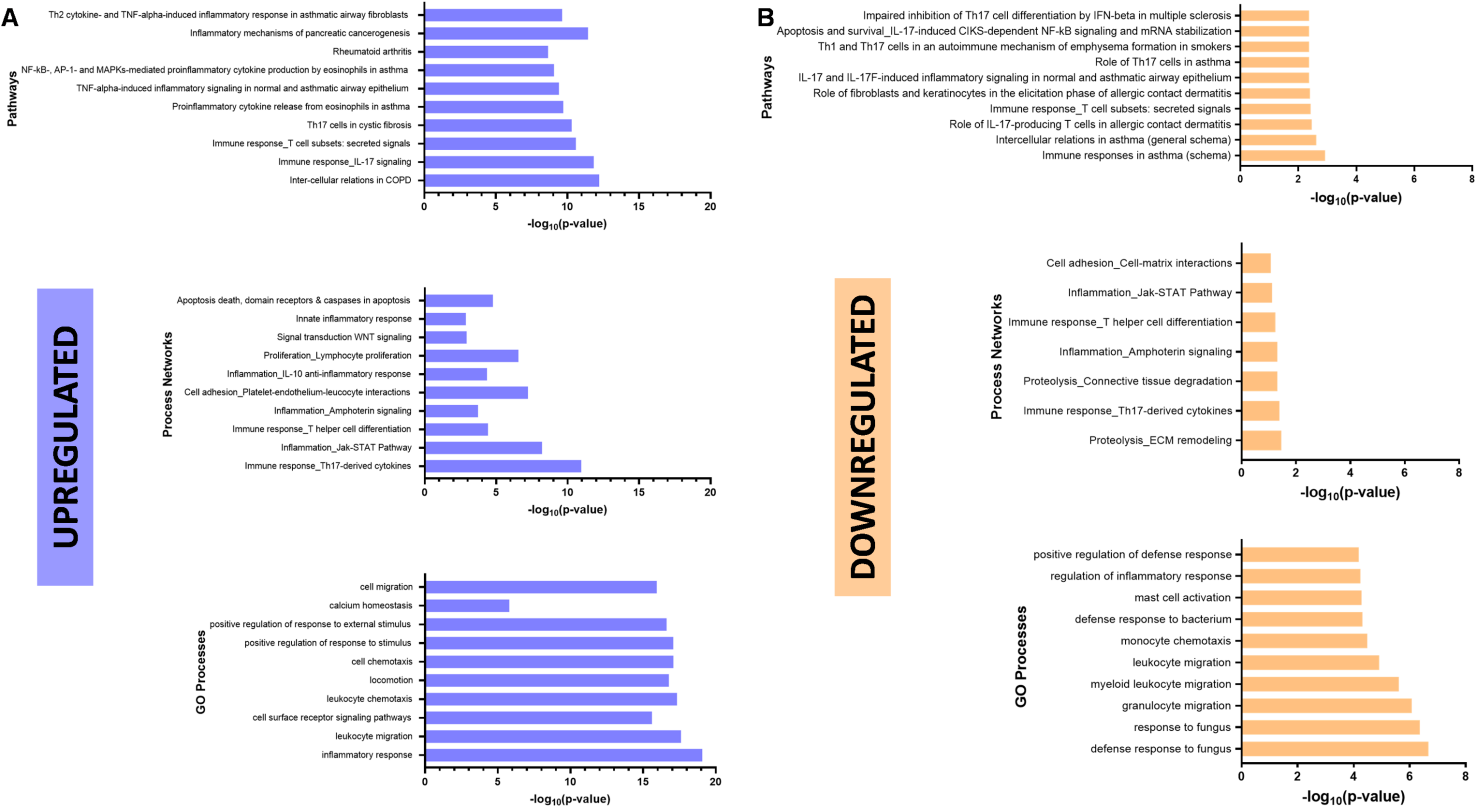
Elexacaftor/tezacaftor/ivacaftor (ETI) impact on canonical pathways. Enrichment analysis was performed in MetaCore (Clarivate 2024) to determine downregulated **(A)** and upregulated **(B)** pathways, networks and processes, using gene set enrichment analysis (GSEA) and the gene ontology (GO) database.

For a closer look at the impact of ETI on the regulation of these pathways, we performed an enrichment analysis on MetaCore. Initially upregulated signaling pathways such as those involving the NF-κB pathway, IL-17 and Th17 cells, were downregulated post-ETI (**Figures 6A, B**, top). ETI therapy resulted in the downregulation of process networks involving the Jak–STAT pathway, the immune response of Th17-derived cytokines, and T helper cell differentiation (**Figures 6A, B**, middle). Lastly, there was a decrease in chemotaxis and migration (**Figures 6A, B**, bottom) of different cell types such as monocytes. Interestingly, processes necessary for response and defense against bacteria and fungus were downregulated after ETI therapy (**Figure 6B**, bottom).

To determine the physiologic effects of these changes in cytokines and other soluble factors, we performed a functional assay that quantitates the net effects of plasma proteins on the activation state of four key transcriptional pathways: NF-κB (reflecting the inflammatory pathway), STAT1 (reflecting the interferon pathway), STAT3 (reflecting the acute phase response pathway), and STAT5 (reflecting hematopoietic and immune cytokines). This integrated functional quantitation (IFQ) assay integrates effects from positive regulators (such as multiple cytokines activating a specific pathway and the presence of activating soluble receptors) and negative regulators (such as antagonizing cytokines or soluble binding proteins). While no consistent effect was seen on the STAT1, STAT3, or STAT5 pathways, there was a strong downward trend in the activating ability of CF plasma post-ETI on the activation of NF-κB (**Supplementary Figure 7A**). This is consistent with the changes detected in the analyses described above.

## Discussion

The highly effective CFTR modulator therapy ETI has provided great clinical benefits to pwCF, yet chronic infections and ongoing inflammatory responses persist (23, 26, 48, 49). ETI partially restores innate immune signaling responsible for bacterial clearance, yet many pro-resolving inflammatory responses remain altered. Individual immune responses to ETI are varied, indicating that not all pwCF benefit similarly from CFTR modulators; particularly, the effects on monocyte and macrophage phenotype and function remain dysregulated (31, 50). In our present study, we identify proteins whose expression was both impacted or unaffected by ETI and had the potential to serve as biomarkers that can be targeted for future therapies.

In the new era of highly effective CFTR modulator therapy, there is much to learn regarding the effects of CFTR modulation on inflammatory markers and their implication in persistent inflammation and dysregulated immune responses. A work from our group previously evaluated cytokine levels and multiple peripheral blood cell populations from pwCF by flow cytometry (51). We found that pwCF prior to ETI therapy had significantly higher levels of IL-6, IL-8, and IL-17A, but low IL-13 when compared to NCF. After 6 months post-ETI, these individuals’ elevated cytokine levels decreased significantly and were comparable to NCF (51). In agreement with this, our current analysis demonstrated that IL-17A significantly decreased post-ETI with an expression profile similar to NCF (**Figures 2B**, **4D**). Supporting our results, the findings by others showed that IL-17A is significantly high in the sputum of pwCF experiencing a *P. aeruginosa* infection (52, 53) and is associated with neutrophilic inflammation, activation of macrophages, and lung function decline. Studies in mice showed that when *P. aeruginosa*-infected mice were treated with the anti-IL-17A antibody, *P. aeruginosa*-mediated inflammation was suppressed, the clinical score was improved, and lung inflammation decreased (54, 55). These results attest to the potential of IL-17A as a target for therapy in CF, as previously proposed, with the goal of decreasing chronic inflammation and damage in the lung (56). Our unpaired analysis also indicated a modest impact of ETI on IL-6 (**Figure 2**).

We also showed that IL-17A and IL-6 expression clustered with other proteins involved in the NF-κB signaling pathway such as MMP-10 and EN-RAGE (**Supplementary Figure 6A**). MMP-10 is elevated in the CF lung and has been suggested to have a protective role against bacterial infection by regulating macrophage activation (57). This was demonstrated by the increased susceptibility of MMP-10-deficient mice to infection by *P. aeruginosa* compared to wild-type mice (57). Although we found that MMP-10 was significantly upregulated in CF prior to ETI and decreased after therapy (**Figure 3D**), long-term studies are needed to determine if this effect is maintained. EN-RAGE has been shown to induce the production of IL-6 and IL-8 both necessary for the regulation of monocyte recruitment (58). Previous studies have detected increased levels of EN-RAGE in the plasma and sputum of pwCF (59). However, these levels were found even more increased (100-fold) in the airway fluid such as the sputum and bronchoalveolar lavage fluid (BALF) of individuals with CF-related diabetes (60–62). In agreement with this study observation, we showed that EN-RAGE expression was significantly higher in pwCF prior to ETI when compared to NCF (**Figure 3E**) and decreased post-ETI therapy. In **Supplementary Table 6**, we listed the proteins in our dataset involved in these pathways: S100, IL-17, and calgranulin C (another name for EN-RAGE). These findings may help shed light on why infections persist regardless of ETI therapy. Therefore, EN-RAGE and other members of the S100 protein family can serve as targets of future therapies in pwCF ineligible for modulator therapies.

The NF-κB signaling pathway was one of the most impacted biological processes in the dataset. The NF-κB pathway can be inhibited by the activation of β-catenin, a protein that participates in the Wnt/β-catenin pathway (39). In human lung epithelial cells, β-catenin interacts with WT CFTR but not mutated CFTR (ΔF508) (63). Failure to interact with ΔF508-CFTR results in β-catenin degradation and subsequently the activation of NF-κB, suggesting that CFTR helps to stabilize β-catenin (64). Similar results were observed in biliary epithelial cells (39). β-Catenin is activated by the destruction protein complex, which includes AXIN1 (65). This protein is upregulated in the bronchial epithelium of pwCF and has been associated with liver disease in CF (39). In our dataset, AXIN1 was also significantly increased before ETI in pwCF when compared to NCF (**Figure 3F**). In agreement with this, the NF-κB pathway was downregulated post-ETI (**Figure 6B**, top). This finding was further supported by an independent assay, the IFQ assay, that functionally measures the activation of specific physiologic pathways. This showed a strong suppression of the activation of NF-κB signaling in the plasma from patients following ETI treatment compared with pretreatment samples (**Supplementary Figure 7A**). On the other hand, the Wnt/β-catenin pathway (**Figure 6A**, middle) remained upregulated. However, the precise mechanisms of how these interconnected proteins are regulated by CFTR modulators remain unknown, particularly, some like AXIN1, which remains unaffected by ETI.

The inflammatory protein CCL20 is produced by macrophages and primary airway epithelial cultures and is elevated in BALF from pwCF (66, 67). CCL20 has antimicrobial properties, as it is able to kill Gram-negative bacteria by means of salt sensitivity (66). Moreover, it can recruit CCR6^+^ B cells, T cells, and dendritic cells, initiating the adaptive immune response. ETI had a modest effect on CCL20 expression in animal and human models (68). Similarly, we showed that CCL20 was significantly elevated in pwCF before ETI compared to NCF, with persistent elevations post-ETI (**Figure 3C**). Augmenting the antimicrobial and immunomodulatory roles of CCL20 should be further investigated in pwCF on CFTR modulators.

Our study was limited to blood plasma samples, which does not discriminate cell type source and effect of circulating factors of each donor. Although measuring inflammatory markers in the plasma provides a robust and easily accessible screening method, it only provides information on systemic inflammation and lacks the status of inflammation in the different parts of the lung that may be impacted by local damage or pathogen loads (69). However, capturing systemic responses to ETI may impact long-term disease outcomes in a multi-organ fashion. Planned studies include new lung models of resident and recruited immune cells and how they are impacted by CFTR modulators. We were also limited to a single time point for biological samples. Studies are needed to determine the long-term effects of ETI on inflammatory protein expression. A novel aspect of our CF study population is that it is noticeably younger than many other recent ETI studies (13.9 ± 5.9 years old) (31, 70), yet it was limited to a mostly self-identified White population. On the other hand, it has been shown that lung function and age can influence biomarker levels in human samples (71). Specifically, adults and individuals with lower lung function exhibit higher concentrations of biomarkers in the serum and sputum. In line with this observation, when we compared the proinflammatory protein profiles of CF pre-ETI and CF post-ETI adults or pediatric groups with non-CF adults (non-CF pediatric data were not available), people with CF demonstrated a higher proinflammatory profile. This highlights a limitation in our study and may partly explain the divergence observed between CF and non-CF groups. Future studies should include a varied pool of participants based on race and ethnicity.

In conclusion, we identified several inflammatory proteins and canonical pathways impacted by ETI therapy, as well as inflammatory profiles unaltered by ETI. We propose IL-17A, MMP-10, and EN-RAGE as candidates for further investigation as potential biomarkers or therapeutic targets. Particularly, further study of EN-RAGE and other S100 proteins in CF can provide a better understanding of persistent infection post-ETI. This research provides a starting point to identify protein expression patterns of interest for biomarker discovery and persistent immune dysregulation in CF, with the goal of improving outcomes for all pwCF.

## Data Availability

The original contributions presented in the study are publicly available. This data can be found here: https://doi.org/10.6084/m9.figshare.28255481.v1.
