# Supplementary material for: Proteomics profiling of inflammatory responses to elexacaftor/tezacaftor/ivacaftor in cystic fibrosis
Source: Front Immunol. 2025 Jan 28;16:1486784. doi: 10.3389/fimmu.2025.1486784 (PMC11811078; doi:10.3389/fimmu.2025.1486784)
Supplement: Supplementary file 1 [file Supplementaryfile1.docx]

**Supplemental Material**


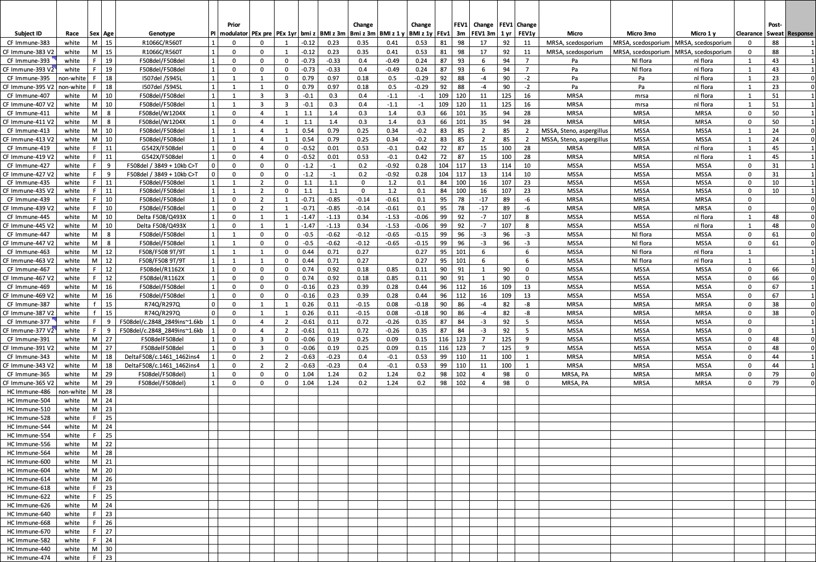


Supplemental Table 1. Deidentified metadata of studied population.


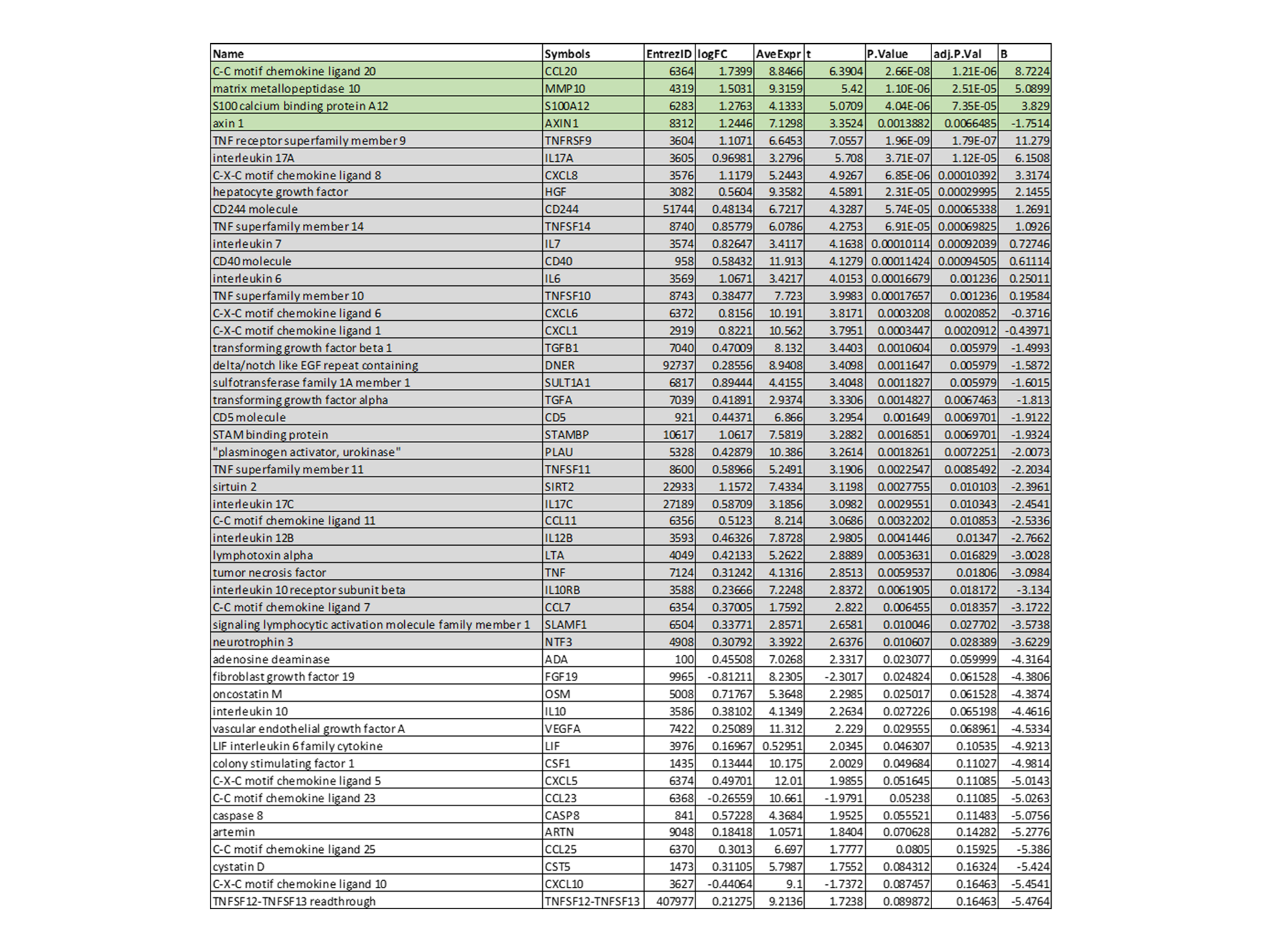


Supplemental Table 2. Unpaired analysis of Non-CF and CF before ETI (CF-Pre). Highlighted in gray are proteins that have an adjusted p ≤ 0.05 and highlighted in green are proteins that were significantly impacted by ETI (p ≤ 0.05 and log_2_ FC ≥ 1.2).

Supplemental Table 3. Unpaired analysis of Non-CF and CF after ETI (CF-Post). Highlighted in gray are proteins that have an adjusted p ≤ 0.05 and highlighted in green are proteins that were significantly impacted by ETI (p ≤ 0.05 and log_2_ FC ≥ 1.2).


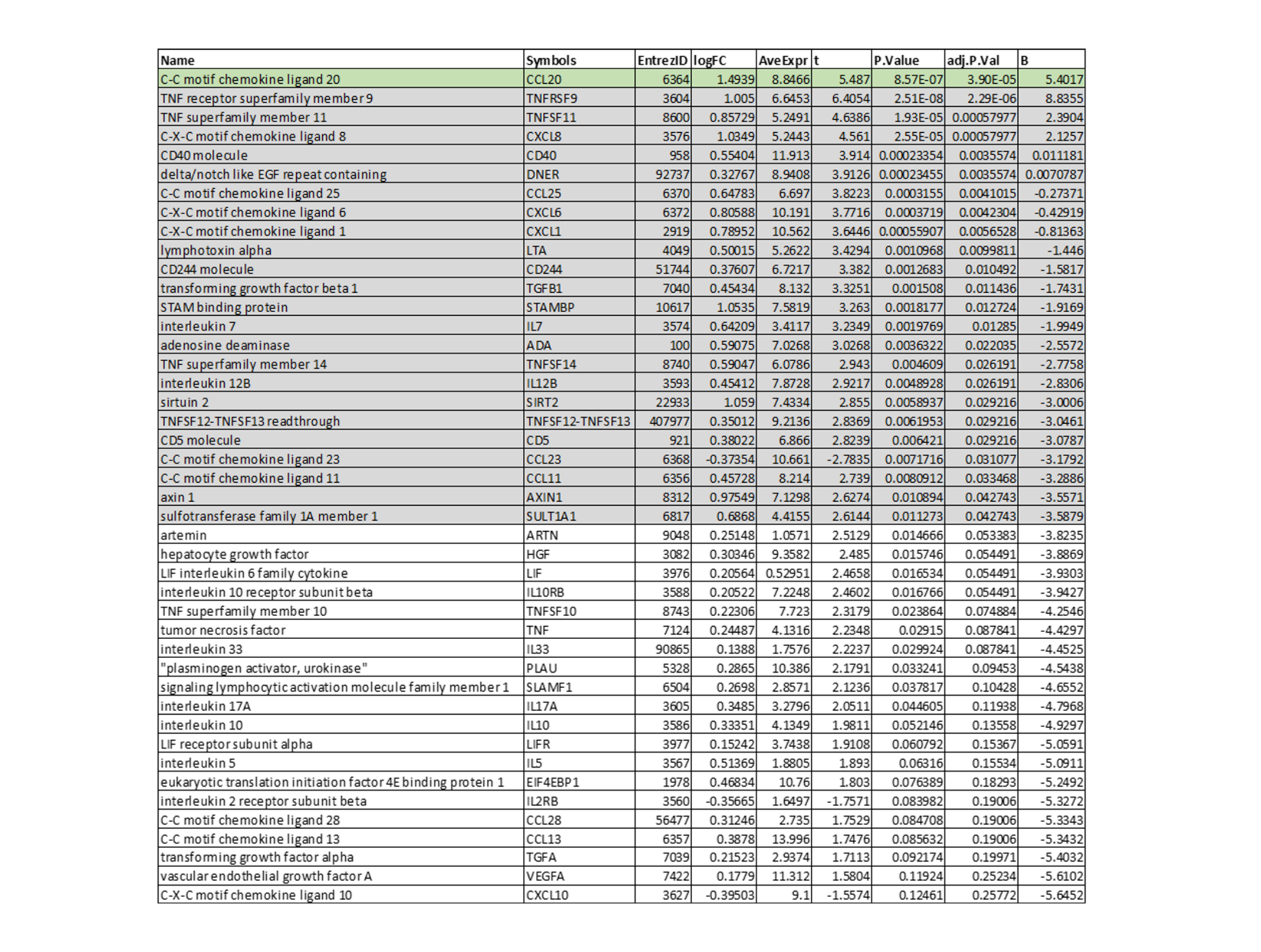


Supplemental Table 4. Paired analysis of CF before ETI (CF-Pre) and CF after ETI (CF-Post). Highlighted in gray are proteins that have an adjusted p ≤ 0.05 and highlighted in green are proteins that were significantly impacted by ETI (p ≤ 0.05 and log_2_ FC ≥ 1.2).


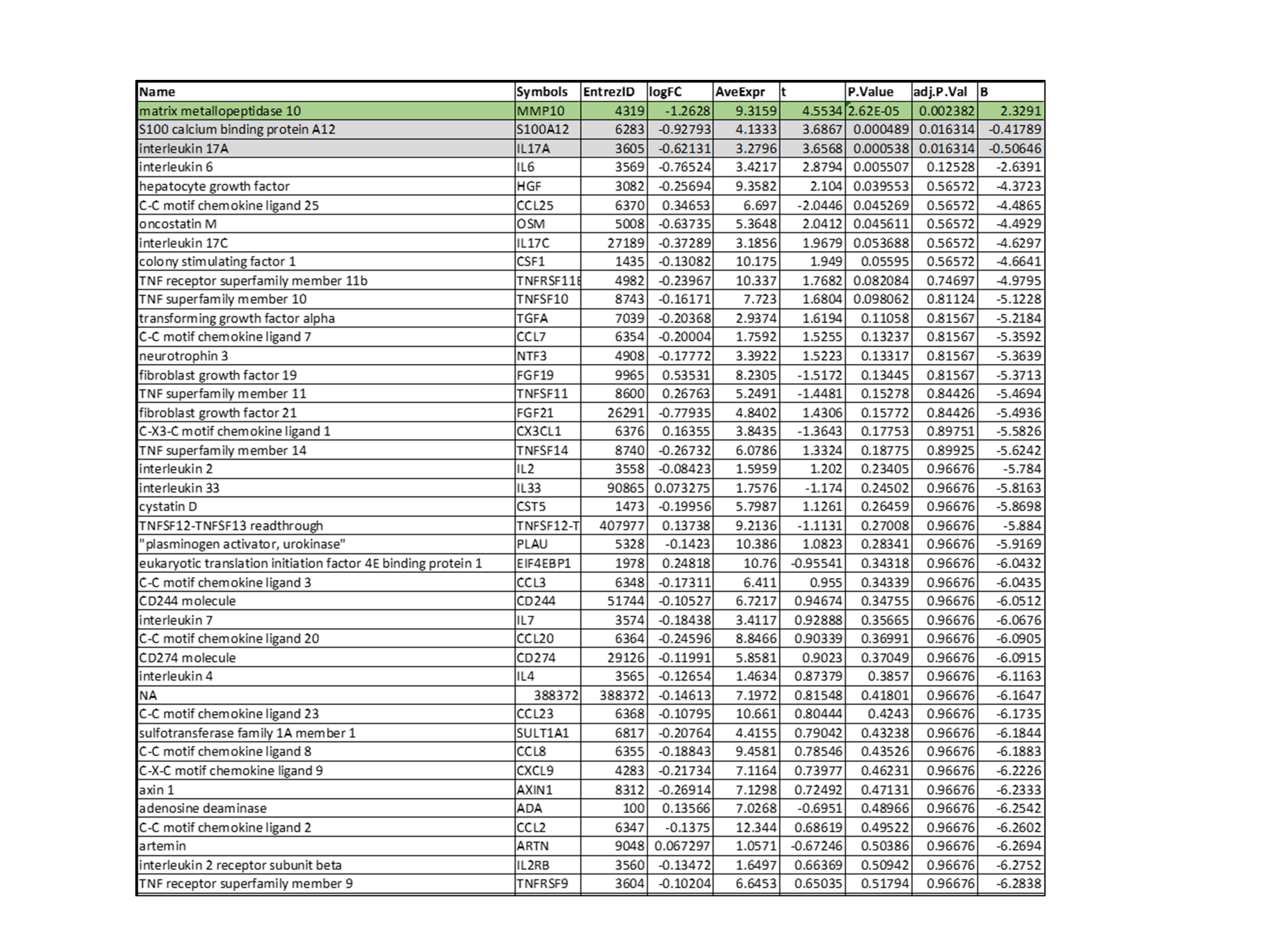


Supplemental Table 5. List of significantly enriched pathways with significance and protein count after unpaired analysis of NCF vs CF-Pre or CF-Post.


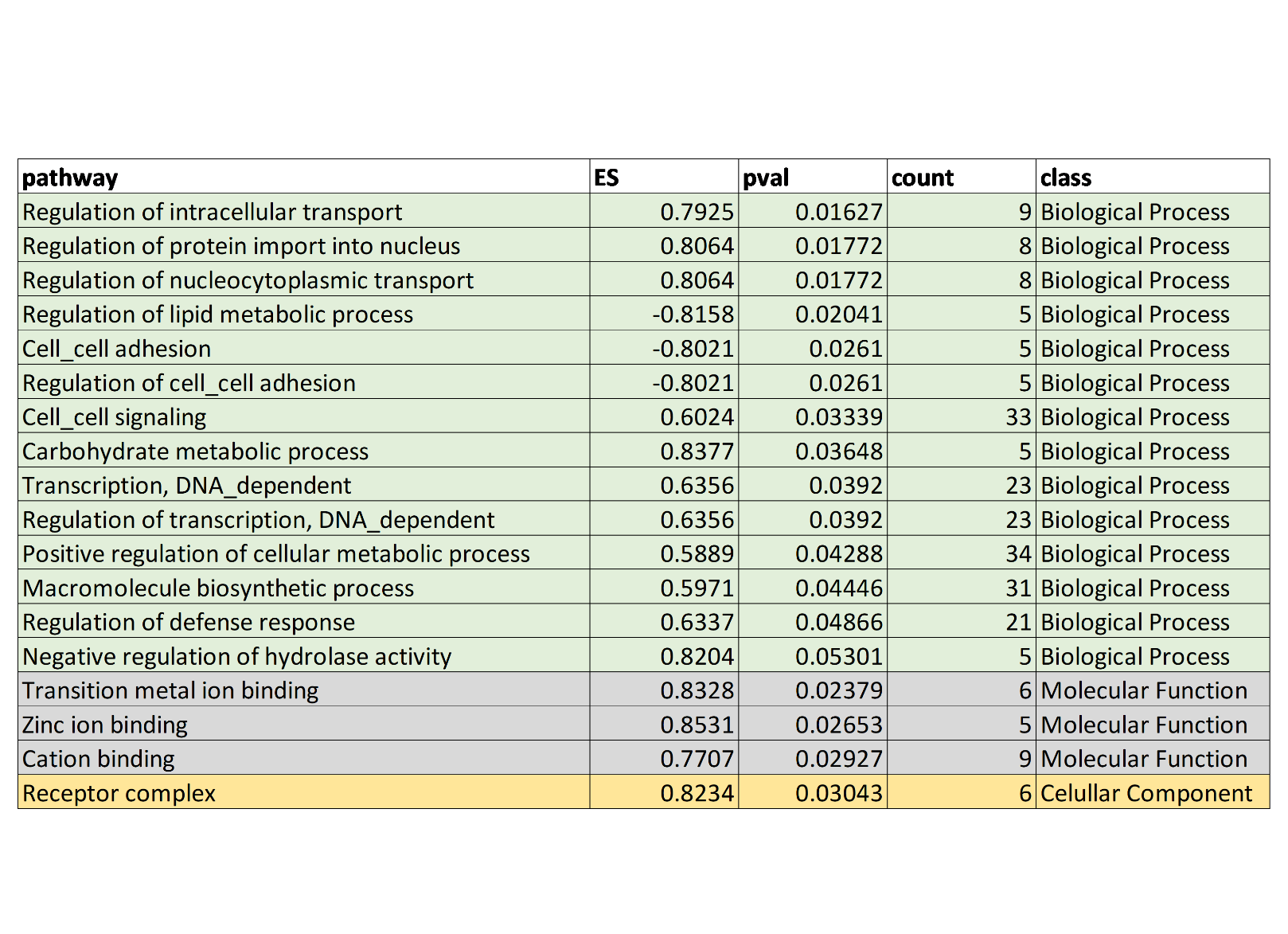


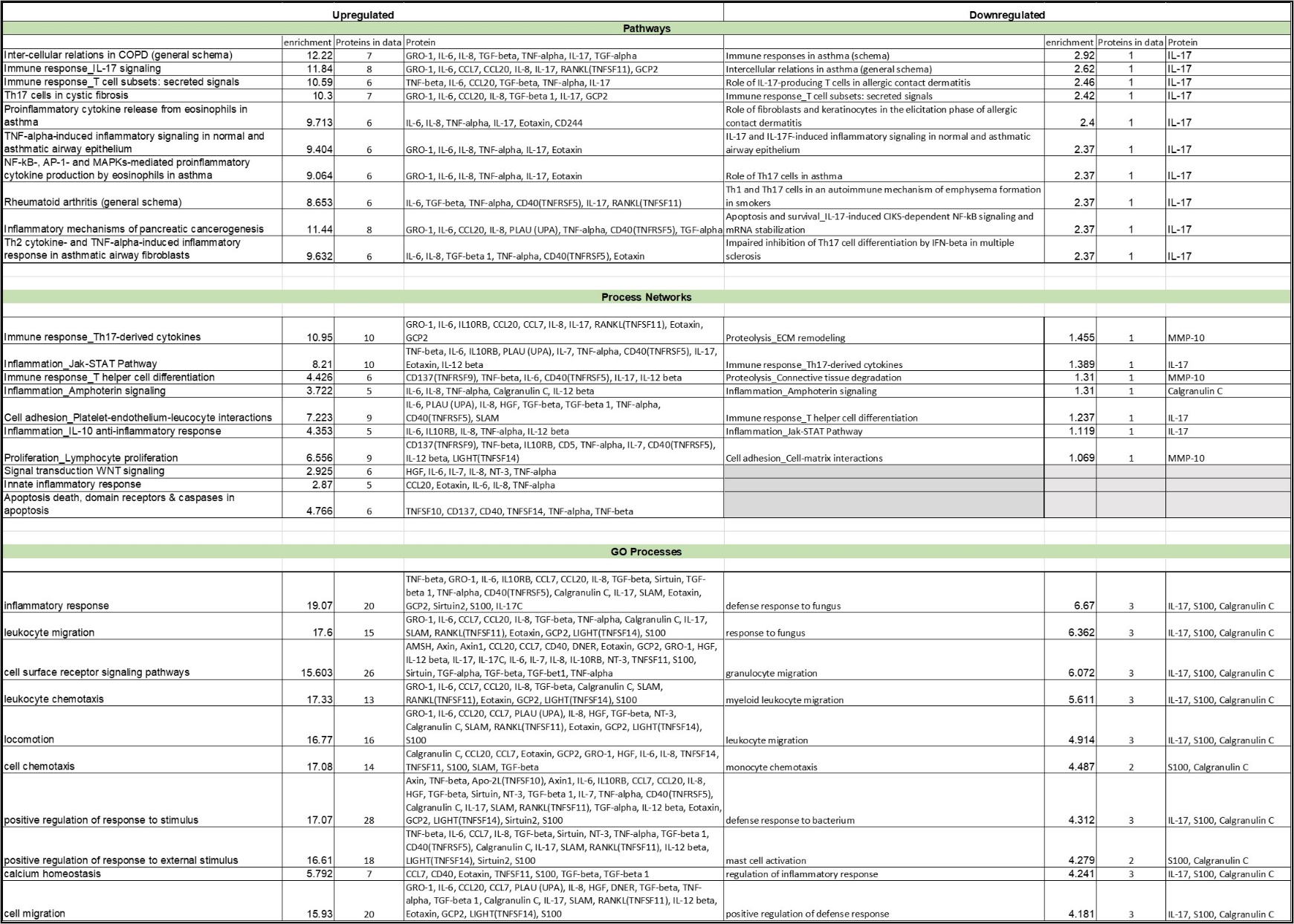


Supplemental Table 6. Upregulated and downregulated pathways, process networks and GO processes. Listed with number of proteins in data, which are part of listed processes.


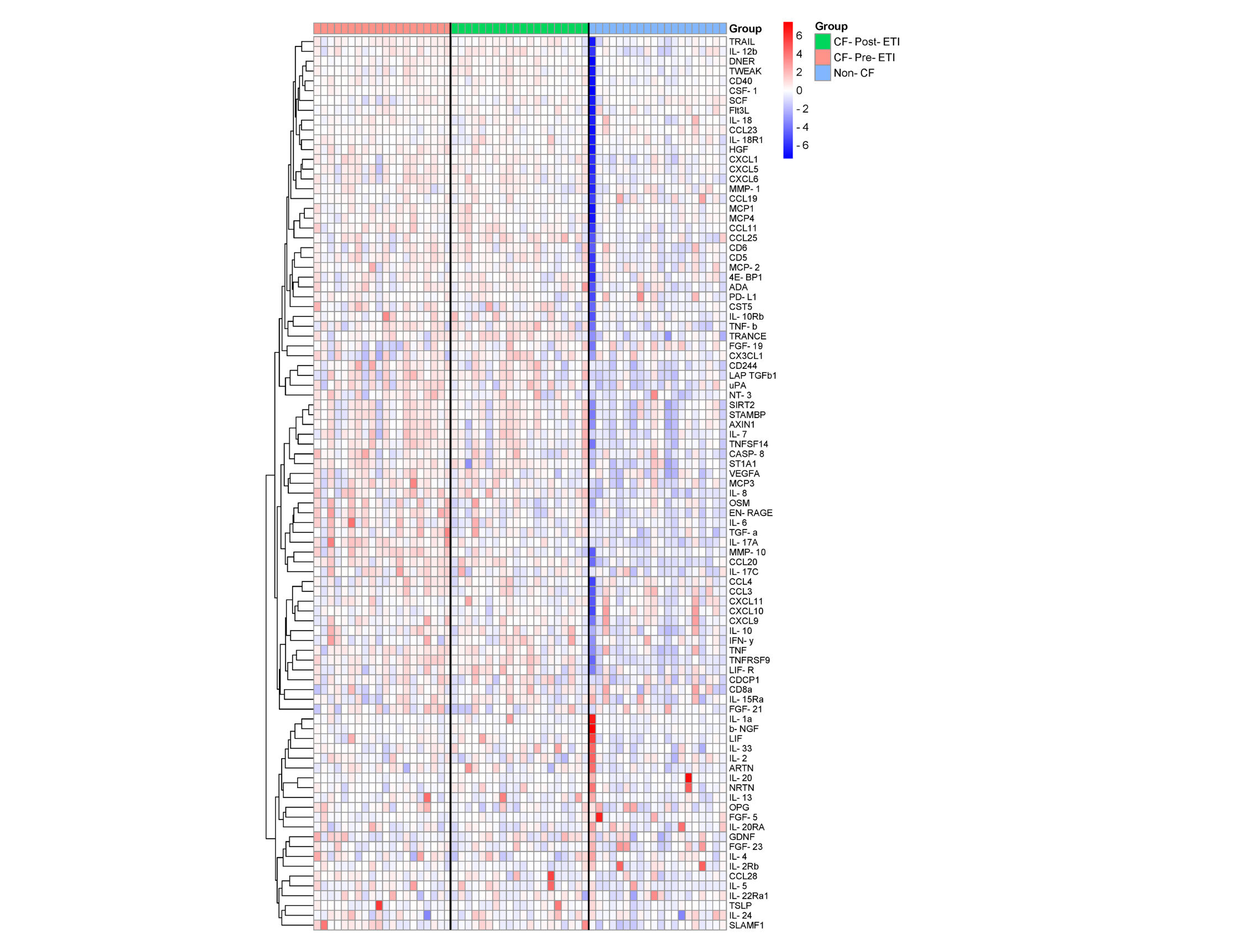


Supplemental Figure 1. Protein expression profiles of inflammatory proteins. Full heatmap of abundance variation profile for each group, by hierarchical clustering with complete linkage, distance determined by the Euclidean method.


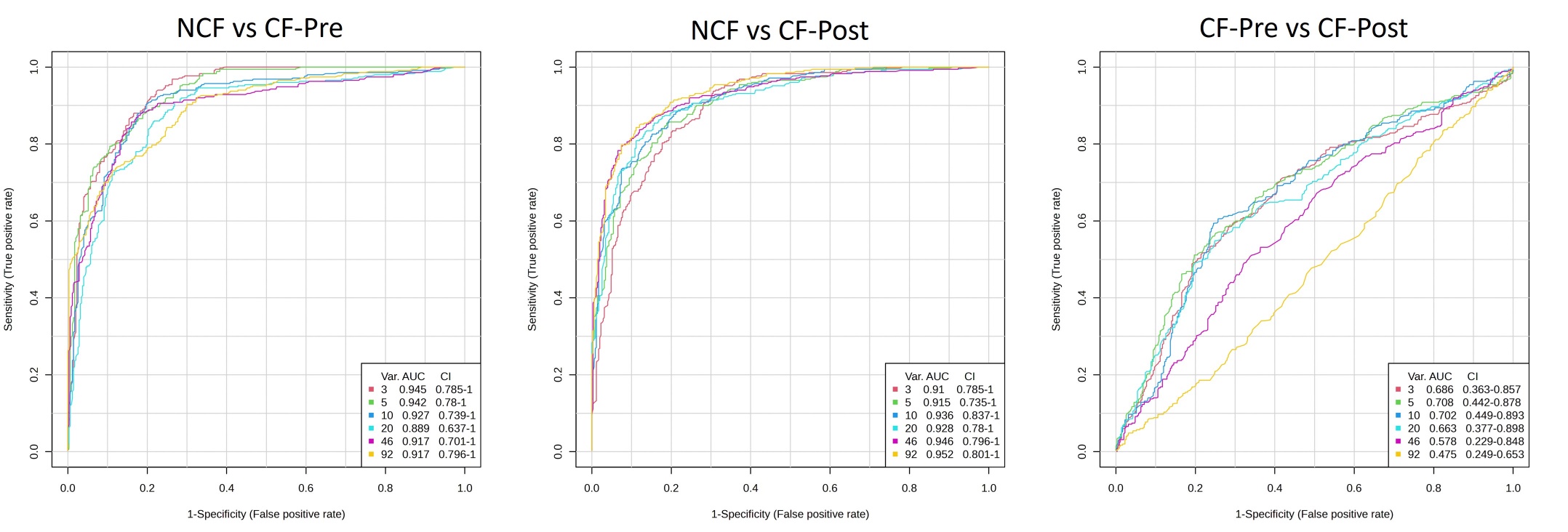

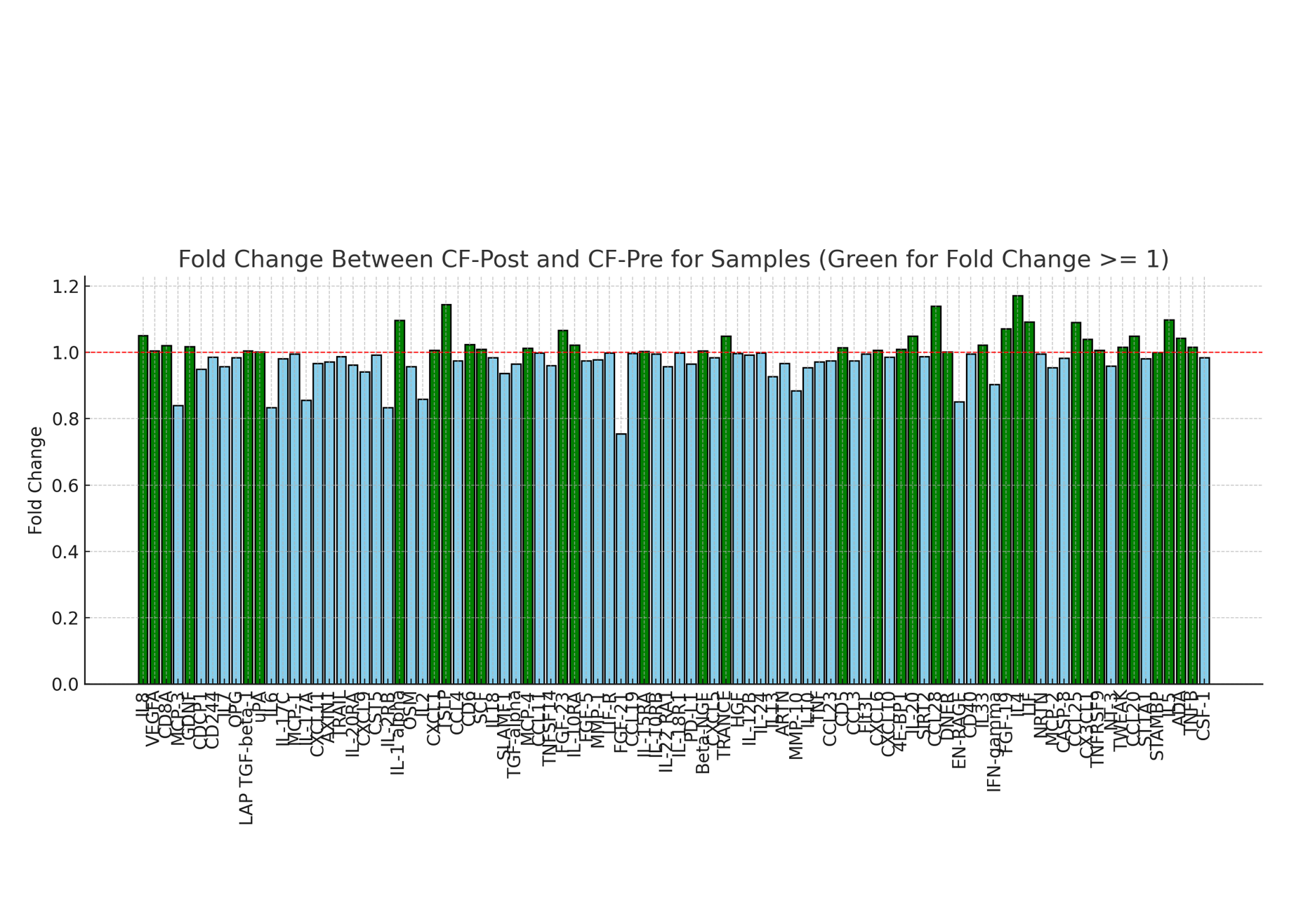


Supplemental Figure 2. ROC plots for each pairwise comparison based on the cross-validation performance. All models tested with indicated number of features tested per model, area under the curve (AUC) and confidence intervals (CI).

Supplemental Figure 3. Fold change of inflammatory proteins in pwCF that cleared bacteria infections.


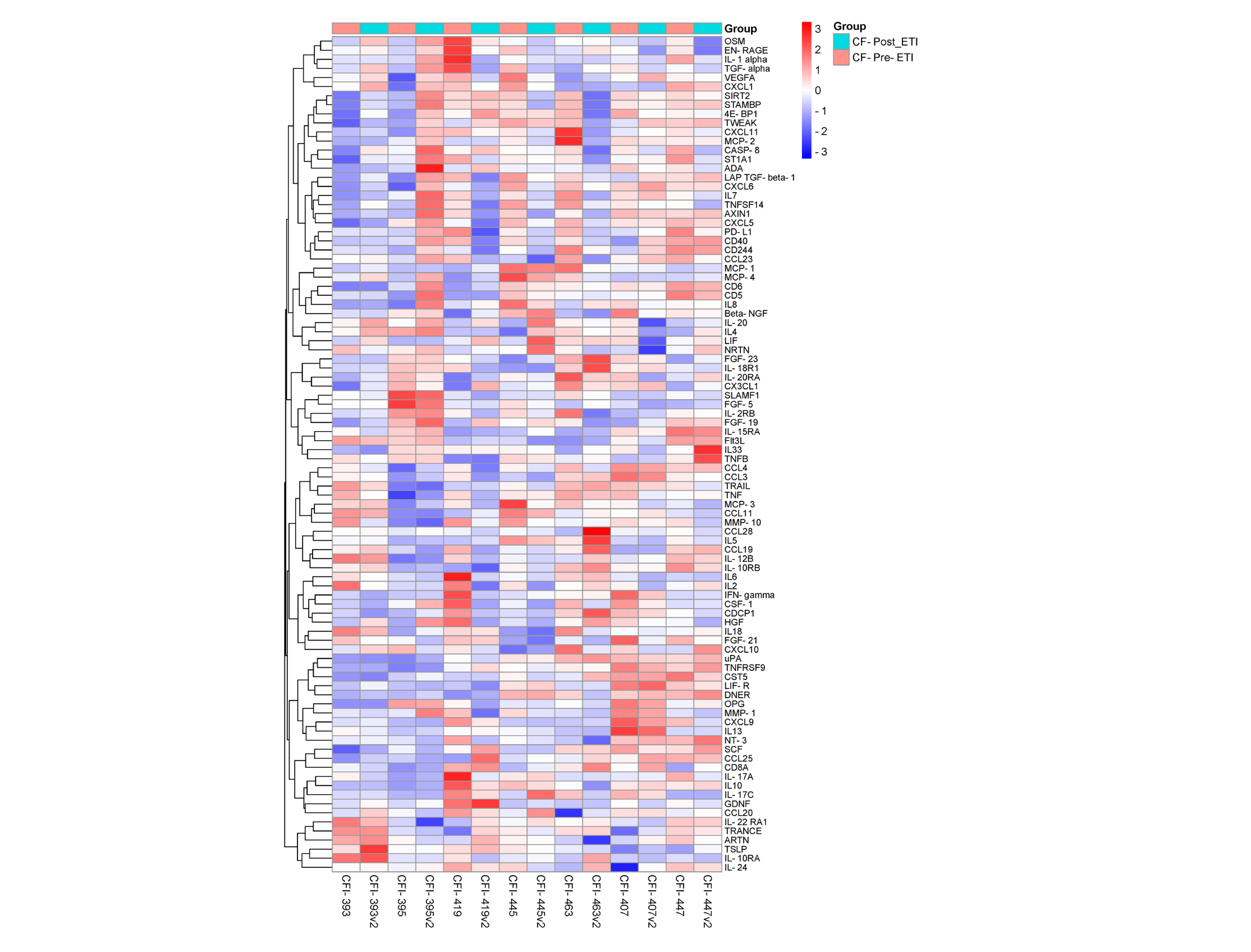


Supplemental Figure 4. Protein expression profile of inflammatory proteins in pwCF that cleared bacteria infections.


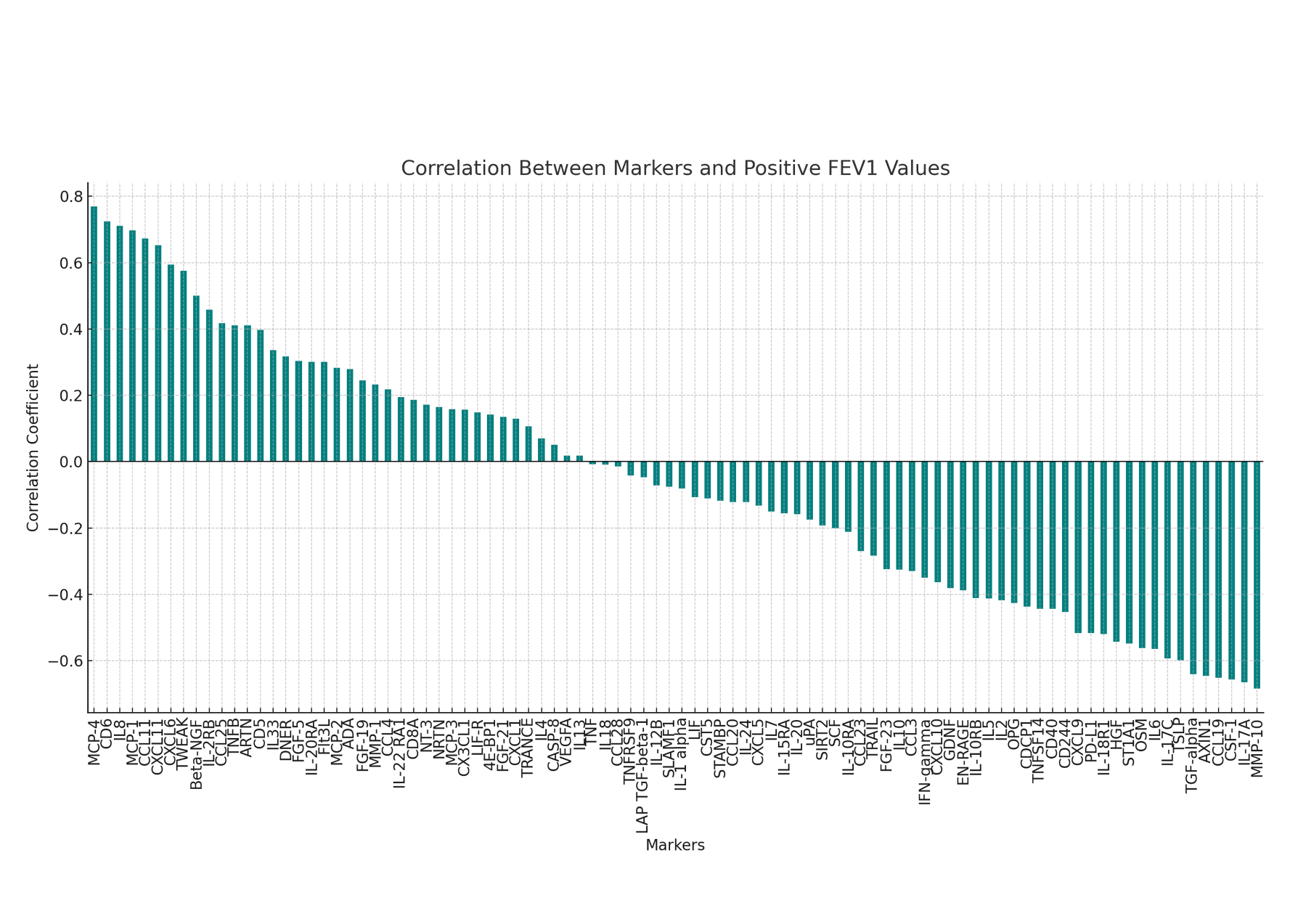


Supplemental Figure 5. Increased FEV1 negatively correlated with expression of MMP-10 and IL-17A.


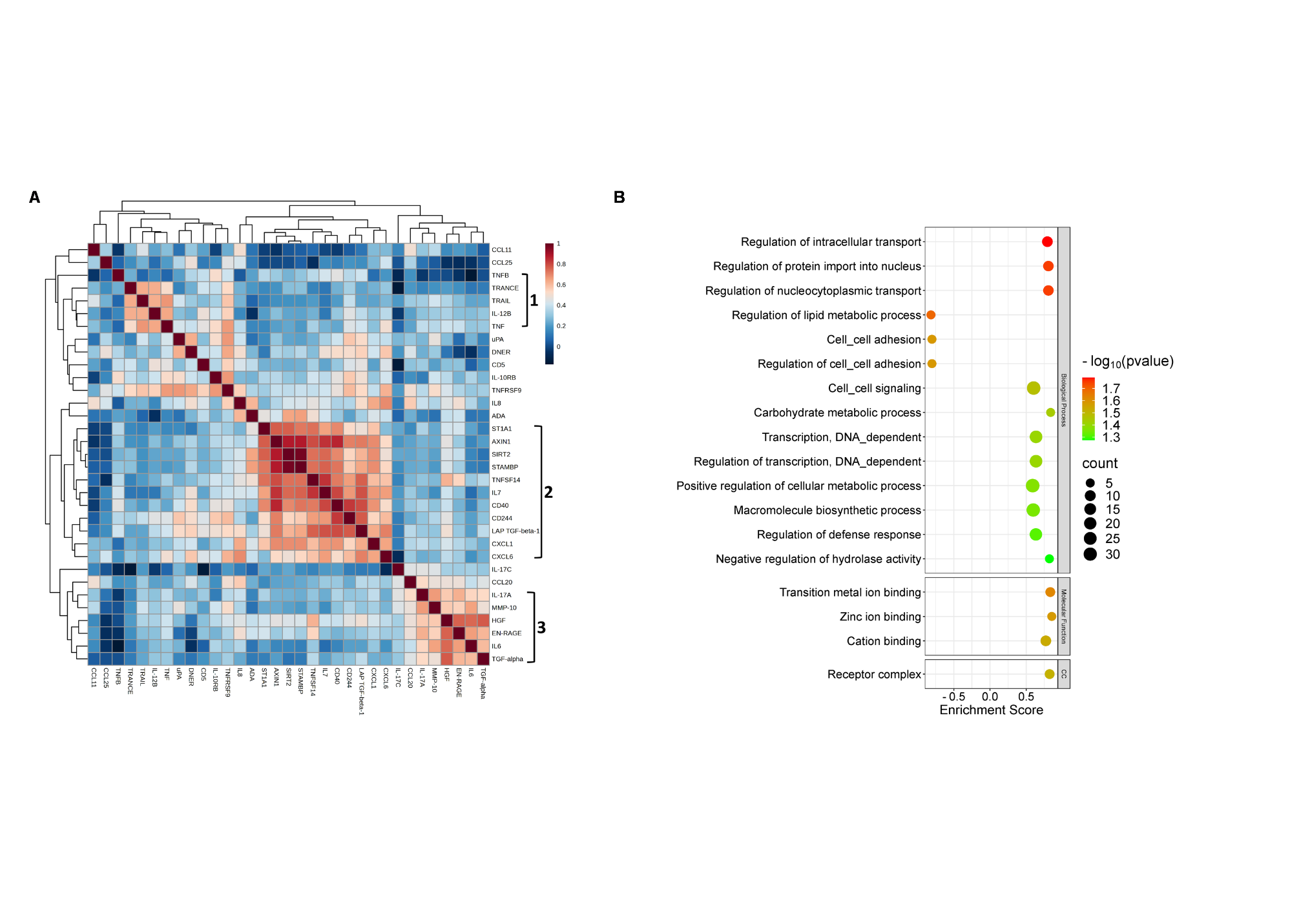


Supplemental Figure 6. Protein expression correlation and significantly enriched pathways. A) Correlation matrix of protein expression profile among proteins evaluated in people with cystic fibrosis (pwCF) using a Spearman rank correlation analysis within MetaboAnalyst (v. 3.0). Correlation coefficient of protein expression from 1 (positive = dark red) to 0 (negative = dark blue). B) Significantly enriched pathways were identified using gene set enrichment analysis (GSEA) and gene ontology (GO) database. Size of the clusters represents the number of proteins involved in the pathway and color represents different threshold of the p-value.


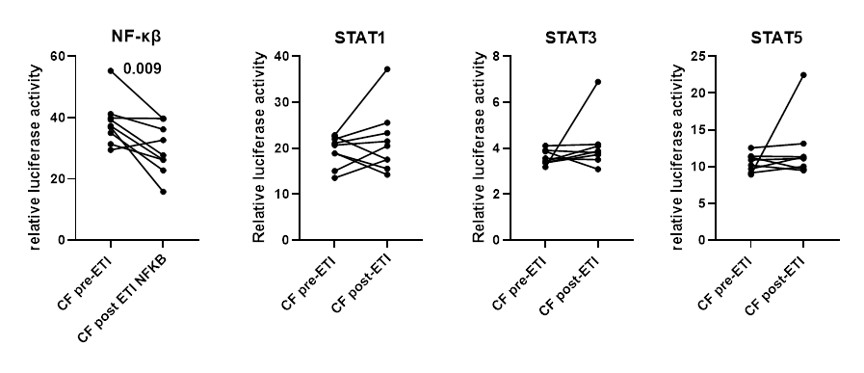


Supplemental Figure 7. Integrated Functional Quantitation (IFQ) assay of modulation of transcriptional pathways. Plasma from people with CF (pwCF) prior to or following ETI treatment were incubated with the indicated reporter cell line, and effects on the specified transcription factor were quantified.
